# Rhinovirus Outbreaks in Long-term Care Facilities, Ontario, Canada

**DOI:** 10.3201/eid1609.100476

**Published:** 2010-09

**Authors:** Jean Longtin, Alex Marchand-Austin, Anne-Luise Winter, Samir Patel, Alireza Eshaghi, Frances Jamieson, Donald E. Low, Jonathan B. Gubbay

**Affiliations:** Author affiliations: Ontario Agency for Health Protection and Promotion, Toronto, Ontario, Canada (J. Longtin, A.L. Winter, S. Patel, A. Eshaghi, F. Jamieson, D.E. Low, J.B. Gubbay);; Public Health Agency of Canada, Toronto (A. Marchand-Austin)

**Keywords:** Rhinovirus, outbreak, fatalities, long-term care facility, viruses, dispatch

## Abstract

Diagnostic difficulties may have led to underestimation of rhinovirus infections in long-term care facilities. Using surveillance data, we found that rhinovirus caused 59% (174/297) of respiratory outbreaks in these facilities during 6 months in 2009. Disease was sometimes severe. Molecular diagnostic testing can differentiate these outbreaks from other infections such as influenza.

Respiratory tract illnesses are a major cause of illness and death among elderly persons, especially those in long-term care facilities. Although the most commonly identified viruses have been influenza virus and respiratory syncytial virus (RSV) (*1*), human rhinovirus (HRV) is being increasingly associated with severe respiratory disease and outbreaks in these facilities (*2**–**6*). Clinical diagnosis of HRV by immunofluorescence and virus culture has been difficult because these methods are unreliable (*7**,**8*). Moreover, because multiple serotypes of HRV exist, retrospective serologic testing cannot be used to evaluate the prevalence of HRV disease (*5*). As a result, the number of outbreaks caused by HRV in long-term care facilities, and the associated illness and death, may be substantially underestimated. We therefore used 2009 surveillance data to estimate prevalence of HRV disease in long-term care facilities.

## The Study

Using data from an active surveillance network, we investigated all respiratory outbreaks (as defined by the Ministry of Health) (*9*), in long-term care facilities, reported from July 1 through December 31, 2009, in the province of Ontario, Canada. The number and timing of specimens collected was left to the discretion of the attending physicians. The regional clinical laboratories cultured specimens (blood, urine, and sputum) for bacteria and performed rapid viral antigen testing for influenza A/B and RSV. The Ontario Public Health Laboratory performed reference microbiology testing that included *Legionella* culture, *Mycoplasma/Chlamydophila* nucleic acid testing (NAT), and virus cultures. Multiplex NAT (Luminex xTAG Respiratory Viral Panel; Luminex Diagnostics, Toronto, Ontario, Canada) was used according to the manufacturer’s recommendations to test nasopharyngeal swabs for viral pathogens (adenovirus, influenza A/B, parainfluenzae 1–4, RSV A/B, enterovirus [ENT]/HRV, coronavirus OC43/229E/NL63/HKU1, and metapneumovirus). An assay specific for pandemic (H1N1) 2009 virus was also performed.

To facilitate turnaround time during periods of higher demand, we used an alternate multiplex NAT kit (Seeplex RV; Seegene USA, Rockville, MD, USA) in conjunction with the Luminex assay. Because the Luminex assay cannot differentiate between ENT and HRV, we used the Seeplex RV kit, which can identify HRV, to confirm results in a random subset of ENT/HRV-positive samples. To type the HRV implicated in outbreaks during which deaths occurred, we amplified and sequenced the hypervariable region of the 5′ noncoding region, the entire viral capsid protein (VP) 4 gene, and the 5′ terminus of the VP2 gene; we then constructed phylogenetic trees as described (*10**,**11*).

During the surveillance period, 297 respiratory disease outbreaks in long-term care facilities were reported to the Ontario Public Health Laboratory; we received samples from 269 facilities (Table 1). A total of 987 specimens were tested (average 3.7 samples/outbreak). Of the 234 (79%) outbreaks for which a pathogen was identified, 174 (59%) pathogens were determined to be ENT/HRV (representing 531 positive samples) and were temporally spread throughout the surveillance period. Pandemic (H1N1) 2009 virus and parainfluenza-1 virus represented 7% and 6%, respectively, of identified pathogens. Other viruses were identified for <2% of outbreaks. Viral co-infection was identified in 7 samples from 7 outbreaks. A subset of 66 samples, representing 15% of ENT/HRV outbreaks, were randomly selected and subsequently tested with the Seeplex RV kit to further differentiate ENT from HRV; HRV was detected in 100% of these specimens.

**Table 1:** Viruses identified in 297 respiratory illness outbreaks in long-term care facilities, Ontario, Canada, July 1–December 31, 2009*

Virus	Outbreaks, no. (%)
Enterovirus/rhinovirus	174 (59.0)
Influenza A	22 (7.0)
Parainfluenza 1	18 (6)
Parainfluenza 2	3 (1.0)
Parainfluenza 3	3 (1.0)
Parainfluenza 4	2 (0.7)
Metapneumovirus	2 (0.7)
Influenza B	1 (0.3)
Respiratory syncytial virus A	1 (0.3)
Respiratory syncytial virus B	1 (0.3)
Adenovirus	0
No specimens received	28 (9.0)
Negative	63 (21.0)

*In 187 outbreaks, 1 virus was detected; in 17 outbreaks, 2 viruses were detected; and in 3 outbreaks, 3 viruses were detected.

Deaths were potentially associated with ENT/HRV in 4 facilities (outbreaks A–D; Table 2). Samples from patients involved in these outbreaks were confirmed to contain HRV; no other causative bacteria or viruses were identified. Clinical data were available for 7 of 13 of the patients who died; 6 deaths were attributed to pneumonia/respiratory infection. Of the 7 patients who died, 5 (71%) had osteoarthritis, 4 (57%) had cardiovascular conditions, 4 (57%) had dementia, 2 (29%) had diabetes, and 1 (14%) had cancer. The only postmortem lung tissue specimen collected was positive for HRV-C (outbreak D).

**Table 2:** Epidemiologic data for rhinovirus outbreaks associated with deaths in long-term care facilities, Ontario, Canada, July 1–December 31, 2009

Outbreak	No. sick residents/total no. residents (%)	No. deaths	No. sick staff members/total no. staff members (%)	Outbreak duration, d	HRV species and strain*
A	28/59 (47)	1	16/80 (20)	38	HRV-A 31
B	32/60 (53)	7	21/100 (21)	43	HRV-A 33
C	19/158 (12)	3	1/200 (0.5)	20	HRV-A 82
D	23/115 (20)	2	3/134 (2)	12	HRV-C N7

*HRV, human rhinovirus.

Nucleotide sequences obtained from isolates from outbreaks A, B, C, and D showed homology to HRV-A 31 (92%), HRV-A 33 (93%), HRV-A 82 (91%), and HRV-C N7 (90%), respectively. We performed multiple sequence alignments of the 410 bp of the 5′ untranslated region, VP4/VP2, and VP1 and compared them with 66 published representative HRV sequences. We could not obtain a VP1 sequence from strains isolated during outbreak D. Phylogenic trees were constructed, and the VP4/VP2 region tree showed better discriminatory power than did that of the 5′ untranslated region (Figure). VP4/VP2 sequence identity was >98% within each outbreak. Sequences were deposited in GenBank under accession nos. GU477323–GU477344.

**Figure:**
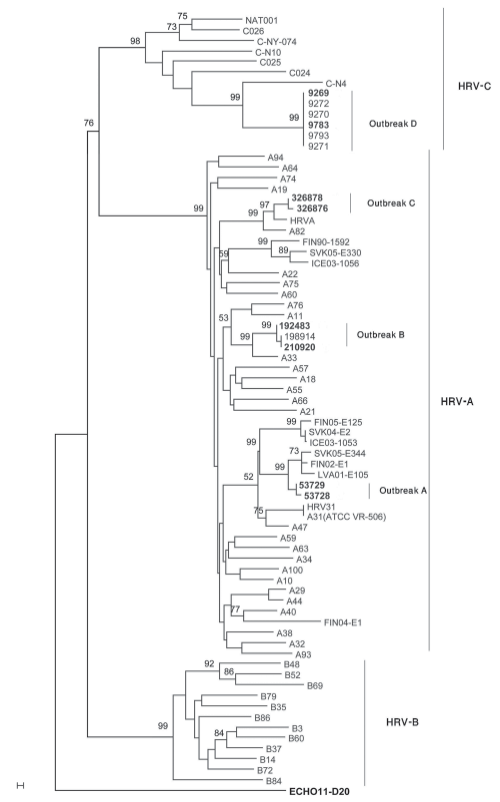
Neighbor-joining phylogenetic tree of human rhinoviruses (HRV) isolated from 4 respiratory disease outbreaks with associated deaths in long-term care facilities, Ontario, Canada. Tree was constructed by using a 549-bp nt region encoding viral capsid protein (VP) 4/VP2, along with strains representative of HRV species A, B, and C. Echo 11 is the outgroup. Bootstrap analysis used 1,000 pseudoreplicate datasets. Scale bar represents 0.1% of nucleotide changes between close relatives. **Boldface** indicates sequences deposited in GenBank.

## Conclusions

We cautiously assume that HRV was the causative organism for 174 (59%) of the 297 respiratory outbreaks in long-term care facilities in Ontario during the surveillance period. Multiplex molecular methods were crucial for rapid identification of the pathogens involved in these outbreaks. We were able to provide results in a timely fashion for every outbreak. However, the cost and expertise associated with such technology might be beyond the reach of some clinical laboratories. Because of the limitations of the surveillance program, we were unable to assess whether such testing is cost-effective in terms of patient care.

Of the 4 outbreaks with associated deaths, 3 were attributed to HRV-A and 1 to HRV-C. The link between respiratory disease severity and HRV-C speciation is debatable (*12*). In a study from Hong Kong, 8 (62%) of 13 adults with HRV-C infection had pneumonia compared with 6 (27%) of 22 adults with HRV-A infection (*13*). However, in the cases we studied, most deaths were associated with HRV-A; a recent study found that HRV-C disease had the same indistinct clinical presentation as did other HRV diseases (*14*).

Viruses isolated from nasopharyngeal swabs by sensitive NAT may represent asymptomatic colonization or nonliving organisms. Although postmortem specimens were available for analysis from only 1 outbreak-related case, we identified HRV in the postmortem lung specimen. Because we do not know whether HRV was present in the lower respiratory tract of the remaining patients who died, a causal association between HRV and severe disease must be made cautiously. We used the 2 NAT assays interchangeably because their reported specificity is >96% for all targets (*15*). Sensitivity for each assay differs according to target; compared with the Luminex assay, Seeplex RV is more sensitive for parainfluenza, RSV, coronavirus, and adenovirus but less sensitive for HRV (*15*). However, despite limitations for epidemiologic data collection, no pathogens other than HRV could explain these outbreaks and associated deaths. Our testing panel did not include human bocavirus or influenza C virus, which could be involved in the remaining 21% of outbreaks that had no identified cause and could even represent confounding factors in the causal relationship of a supposed pathogen.

In conclusion, using data from a routine surveillance network, we found high prevalence of HRV during a period that encompassed the first and second waves of pandemic (H1N1) 2009. These findings are in accordance with the increasing knowledge that HRV outbreaks cause severe and fatal disease.
